# Q&A: What is regeneration, and why look to planarians for answers?

**DOI:** 10.1186/1741-7007-10-88

**Published:** 2012-11-08

**Authors:** Alejandro Sánchez Alvarado

**Affiliations:** 1 Howard Hughes Medical Institute and Stowers Institute for Medical Research, 1000 E. 50th Street, Kansas City, MO 64110, USA

## What is regeneration?

Historically, philosophers, naturalists and biologists alike have referred to the restoration of missing body parts after traumatic injury as regeneration. While still valid today, the concept of regeneration has expanded through the years to include a diverse set of phenomena. For instance, August Weisman considered physiological cell renewal to be regeneration and wrote so in a chapter dedicated to regeneration in his seminal 1893 book *The Germ Plasm*: 'the functions of certain organs depend on the fact that their parts continually undergo destruction, and are then correspondingly renewed. In this case it is the process of life itself, and not an external enemy, that destroys the life of a cell' [1]. Soon after, TH Morgan would also attempt to refine the precision of the concept of regeneration by coining terms that distinguish between regeneration requiring cell proliferation (epimorphosis) and regeneration effected by tissue remodeling (morphallaxis) [2]. Presently, regeneration is used to include multiple restorative processes manifested either as a result of physiological turnover (for example, the renewal of blood, skin and gut epithelial cells) or injury, and more recently has been used to define a branch of medical practice referred to as 'regenerative medicine'. Thus, rather than becoming more specific, the concept of regeneration has become much more general. This peculiarity can be attributed in great part to the fact that presently, and not unlike previous centuries, little unambiguous molecular, cellular, and evolutionary evidence exists to support a common or divergent mechanism controlling physiological and traumatic regeneration within and between species. That such diverse biological phenomena as adult neurogenesis and limb regeneration can be catalogued under the same umbrella is indicative of our limited mechanistic understanding of regenerative processes, and thus underscores how much more discovery research remains to be done.

## Regenerative ability is broadly but unevenly distributed across species; why can't all animals replace tissues and organs after amputation?

**Figure 1:**
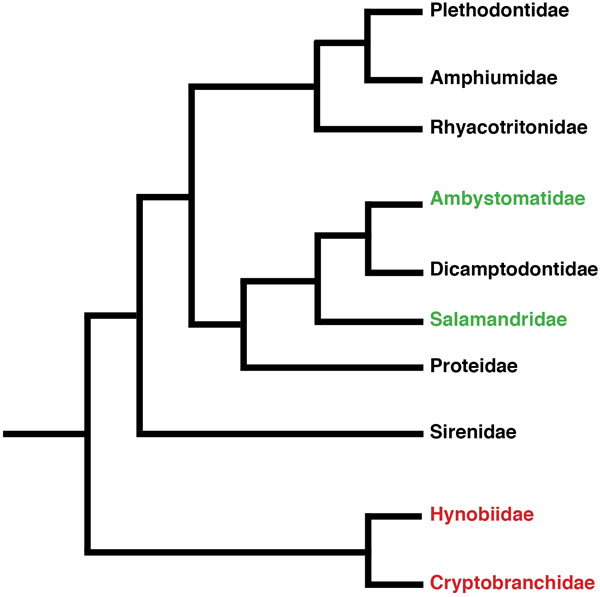
**Phylogenetic relationships of the salamanders**. In green are the species known to possess Prod1 and to display limb regeneration capacities. Red denotes the extant primitive groups of salamanders (see text for explanation). The tree is based on multiple sources [26-28].

A satisfactory explanation to this question is presently lacking. Many organisms known to regenerate body parts after injury have close relatives that have been subjected to similar if not identical selective pressures, and yet are incapable of regeneration [3]. Two possibilities are plausible: 1) the common ancestor to both species had regenerative capacities, but only one descendant retained such properties; and 2) the common ancestor had no such regenerative capacities and that speciation somehow resulted in the acquisition of regenerative properties in one, but not both descendants. Thus, to understand the seemingly random distribution of regenerative properties across animal species, it becomes essential to determine whether regeneration has evolved at a macroevolutionary (above and across species) or at a microevolutionary (within species) level. Brockes and colleagues [4] have recently proposed that limb regeneration in salamanders may have evolved locally in this organism, that is to say at a microevolutionary level. Their hypothesis is based on the observation that the three finger protein (TFP) family member Prod1, a key regulator of both patterning and growth in the regeneration of limbs, is likely unique to the salamanders. While tantalizing, this hypothesis needs to be tested in related, but phylogenetically more primitive, salamanders (Figure 1). If indeed Prod1 arose recently in Salamandridae (newts and salamanders) and Ambystomatidae (Axolotl) evolution (Figure 1, in green) as the result of a local expansion of the TFP family, more basal salamanders such as Hynobiidae and Cryptobranchidae would be expected to lack Prod1 and thus the capacity for limb regeneration (Figure 1, in red). However, there is some evidence that primitive salamanders (Cryptobranchidae) are nevertheless capable of regenerating appendages [5,6]. Still, whether evolution is ancestral or a species-dependent invention is a question that has yet to be conclusively resolved.

## Why is the evolutionary origin of regeneration an important issue?

The emerging field of regenerative medicine aims to identify strategies to repair tissues, organs, and human body parts that cannot be naturally replaced when damaged by either trauma or disease. Examples are spinal cord injury, loss of limbs and the loss of neurons to stroke and degenerative diseases like Parkinson's and Alzheimer's. Given that natural regeneration of such tissues occurs with frequency across vertebrate and invertebrate organisms alike, it stands to reason that if we can understand these processes, we should be able to extrapolate this knowledge to human health matters. If regeneration is evolutionarily ancestral and its mechanisms conserved across all animals with regenerative capacities, then it should be possible to coax mammalian tissues to launch a regenerative response by modulating pre-existing repair and regenerative mechanisms. On the other hand, if regeneration is an attribute invented independently multiple times in evolution, understanding which aspects of this process are unique, species-dependent inventions will also have an impact on how to apply the knowledge derived from animal regeneration studies to human health. For instance, understanding why a particular regenerative process takes place in a model system but not in human tissues may help identify new molecular pathways and cellular activities that could be extended to human cells and tissues to stimulate regeneration should endogenous mechanisms not be readily available. Either way, deciphering the modes and mechanisms driving regeneration in multiple model systems will not only help us resolve a long-standing question in biology and evolution, but also have clear ramifications for our understanding of wound healing and regeneration in humans.

## Why are planarians a good model system to study regeneration?

There are many reasons why we chose planarians as a model system for the molecular and cellular dissection of animal regeneration [7]. Our decision was driven in great part by a need to bridge experimental gaps left exposed by traditional genetic model systems. The pronounced limitations of somatic tissue turnover and regenerative properties in standard invertebrate models such as *Drosophila *and nematodes, coupled with the difficulties of studying adult vertebrate somatic stem cells *in vivo*, were compelling reasons to examine and test the suitability of planarians, free-living members of the phylum Platyhelminthes, to inform both regeneration and stem cell biology. Planarians, which are non-parasitic flatworms, display remarkable regenerative capacities for all of their tissues, irrespective of whether these were derived from endoderm, mesoderm or ectoderm. Because of their evolutionary position, these bilaterally symmetric, triploblastic organisms were expected to share with vertebrates a large number of the molecular and cellular processes that make form and function possible in animals. We now know that this is indeed the case, as planarians share with vertebrates all of the major developmental signaling pathways responsible for the establishment of the bilateral body plan [8,9]. In addition to their remarkable powers of regeneration, and in contrast to vertebrate regeneration model systems, planarians are small (about the size of a toenail clipping), and rather easy and relatively inexpensive to rear in great numbers in the lab, allowing for genome-wide functional studies of regeneration. Planarians were also very attractive as a model system because an extensive body of literature spanning over two centuries exists, which describes in great detail the remarkable developmental plasticity of these animals [8]. This exquisite body of knowledge has, for the most part, just begun to be examined using the rigors and methods of modern molecular and cellular biology.

## Why study one particular species - *Schmidtea mediterranea*?

**Figure 2:**
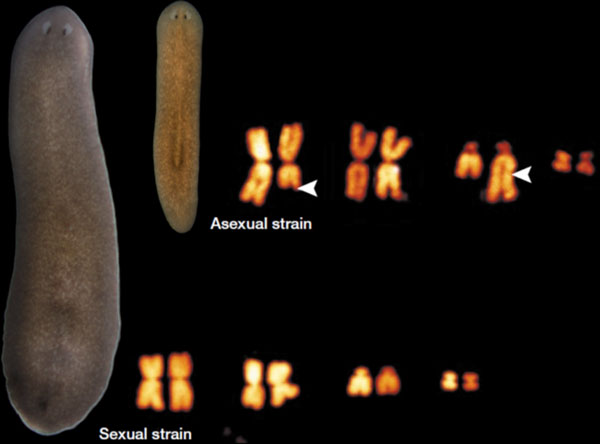
**The planarian *Schmidtea mediterranea***. Sexual (left) and asexual biotypes are shown with their corresponding diploid karyotypes. Modified from [17,22].

**Figure 3:**
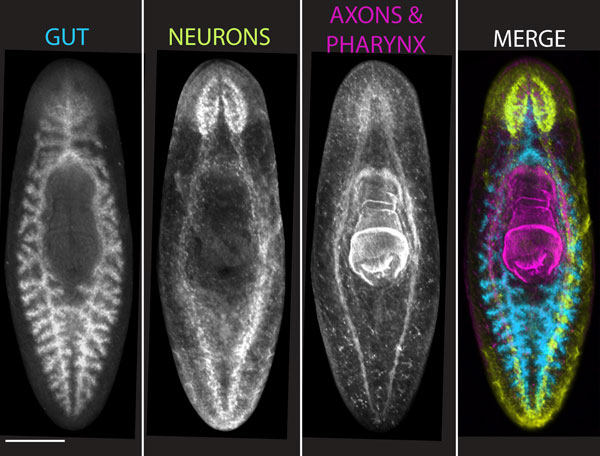
**Sampling of the anatomical complexity displayed by the planarian *S. mediterranea***. Overlay of gut (blue, *Smed-porcn-1*), neurons (yellow, *Smed-PC-2*), axons, and pharynx (magenta, anti-α-tubulin antibody). Scale bar 200 μm. Modified from [8].

This particular species was selected because it met a number of criteria deemed necessary to perform molecular, cellular, and mechanistic studies successfully [7]. First, *S. mediterranea *is a stable diploid possessing four pairs of chromosomes (Figure 2). Second, it has a relatively small genome (approximately 800 Mb or the equivalent of the first four human chromosomes), making it relatively easy to sequence the genome [10]. Third, this species exists in two biotypes - one sexual, the other asexual - allowing for a comparison of both sexual and asexual reproduction and embryogenesis and regeneration. Fourth, because of its robust regenerative capacity, we were able to generate clonal lines that have limited polymorphisms in the population, thus facilitating gene isolation, and spatial and functional assays. Finally, the complex anatomy of planarians is well represented in *S. mediterranea*, allowing us to identify tissue-specific markers and thus define and visualize all organ systems (Figure 3).

## What triggers regeneration?

Across multiple species and phyla, the stimulus for regeneration is amputation. Planarians are no exception. Wounding and amputation in this organism leads to a coordinated cellular and molecular response that can be measured and is currently under intense investigation. We know, for example, that upon amputation, the body wall musculature undergoes depolarization, which in turn results in the contraction of the muscle fibers near the amputation plane, effectively reducing the surface area of the wound. This is followed by a loss of columnar morphology of the epidermal cells adjacent to the wound and their subsequent migration over the wound, until the exposed tissues are completely covered by a monolayer of these cells. This amputation-induced epithelial-mesenchymal interaction is likely involved in the signaling that triggers regeneration, as one of the earliest genes induced in response to wounding is part of the ancient, broadly conserved Wnt/β-catenin signaling pathway [11-13], which also plays a key role in wound healing [14].

## Which types of tissue can regenerate?

All of them - that is why planarians are so attractive for the study of regenerative mechanisms. As such, it becomes possible to study how the differentiated derivatives of all embryonic germ layers (ectoderm, mesoderm and endoderm) can be restored in an adult context after they have been lost to amputation.

## What is the smallest fragment of tissue capable of regenerating a complete worm?

This often-asked question was answered by TH Morgan in 1898 [15]. He reported that a fragment equivalent to 1/279th the size of the original animal was sufficient to produce a complete animal. He arrived at this number by first measuring the animal using eye-micrometers in his microscope, for which each division was 1/53 mm and 1/28 mm. After measuring the worm, Morgan would then draw, cut, and weigh a thin but larger cardboard scale replica of the intact animal. He would then cut the animal into the smallest possible pieces, measure each piece, and then cut an equivalent sized fragment form the cardboard replica. He followed the regeneration of the cut fragments, and then measured the weight of the cardboard pieces corresponding to the animal fragments that completed regeneration successfully. In other words, the cardboard replica was measured to weigh 279.5 mg, and the smallest planarian fragment that could regenerate corresponded to a cardboard piece weighing 1 mg, thus resulting in the 1/279th value for the smallest piece capable of regenerating a complete worm.

## Is some sort of specialized stem cell required for regeneration?

Yes. Large numbers of small, undifferentiated cells populating the body plan of many flatworms were noticed towards the end of the 19th century [8]. These cells were also noted to be mitotically active, and their role in regeneration was confirmed by the pioneering work of Bardeen and Baetjer [16]. These investigators reported in 1904 that animals exposed to ionizing radiation lost their regenerative capacities. When the worms were inspected histologically, Bardeen and Baetjer reported a complete absence of both mitotic activity and undifferentiated cells. These specialized cells are referred to as neoblasts.

## What are neoblasts?

Neoblasts are pluripotent, somatic stem cells that are broadly distributed across the planarian anatomy. In asexual animals they are the only cells capable of undergoing cell division and as such can be readily eliminated by gamma-irradiation to produce an animal that can survive for several weeks, but is incapable of mounting a regenerative response upon wounding. Neoblasts are small (approximately 5 µm in diameter) and by morphology alone correspond to approximately 25% of all cells in the organism. They share with other stem cells the characteristic of having a large nucleus containing highly decondensed chromatin and a scant, basophilic cytoplasm [17]. Molecular markers and genes affecting the function of neoblasts and their progeny have been identified [18-20], providing the field with novel molecular tools to characterize their biological functions *in vivo*.

## Can a single neoblast generate a whole animal?

While the *in vitro *culture of neoblasts has yet to be established, single stem cell transplantation into adult planarians is possible, making the animal itself a tissue culture chamber in which to grow these cells. Recent experiments have unambiguously demonstrated that, with some frequency, single, transplanted neoblasts can restore viability and rescue many of the morphological defects of lethally irradiated adult animals [21]. Interestingly, under these conditions, the rescue of the irradiated animals occurs through a clonal expansion rather than migration of the injected cell, followed by expansion of the resulting colony of stem cells. These data would indicate that neoblasts are not migratory cells, a somewhat surprising result given how many niches (that is, the cellular microenvironment capable of supporting the maintenance of stem cells in plants and animals) were left vacant by the irradiation that would have been expected to promote stem cell mobilization.

## Can neoblasts migrate?

**Figure 4:**
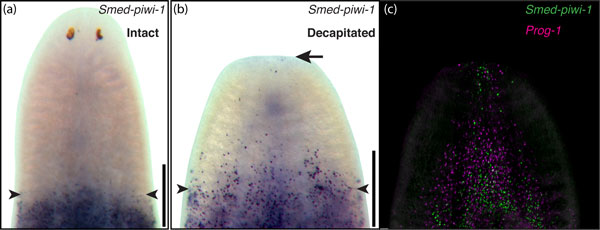
***In vivo *migration of stem cells in planarians**. **(a) **Neoblasts labeled with the stem cell marker *Smed-piwi-1 *(purple) in a partially irradiated, unamputated (intact) animal. **(b) **Migrating neoblasts in a decapitated, partially irradiated animal. Arrow points to neoblasts at or near the site of amputation. **(c) **A decapitated, partially irradiated animal in which cells are visualized via fluorescent *in situ *hybridization. Neoblasts are in green (*Smed-piwi-1*) and post-mitotic progeny in magenta (*Prog-1*). (a,b) Arrowheads denote the boundary between irradiated (top) and unirradiated (bottom) tissues. Modified from [22].

Recently, we have shown that neoblasts can in fact migrate, but appear to do so only when a breach in structural integrity such as amputation is inflicted upon the animal. This stem cell behavior was discovered by selectively eliminating stem cells from only parts of the animal with gamma-irradiation. Essentially, the trunks of animals were protected from irradiation by a lead shield, while the head and tail were subjected to lethal doses of irradiation. When the animal is not amputated, the stem cells residing in the protected region do not mobilize to repopulate the irradiated tissues (Figure 4a). However, if the partially irradiated animal is then decapitated, a marked mobilization of neoblasts towards the wound site becomes readily apparent (Figure 4b,c) [22]. The fact that neoblasts do not appear to migrate in the absence of amputation, while at the same time continuing to effect tissue homeostasis [21], indicates that different mechanisms for restorative versus injury induced regeneration are likely to exist.

## Can the regenerative behavior of cells be traced to gene function in planarians?

**Figure 5:**
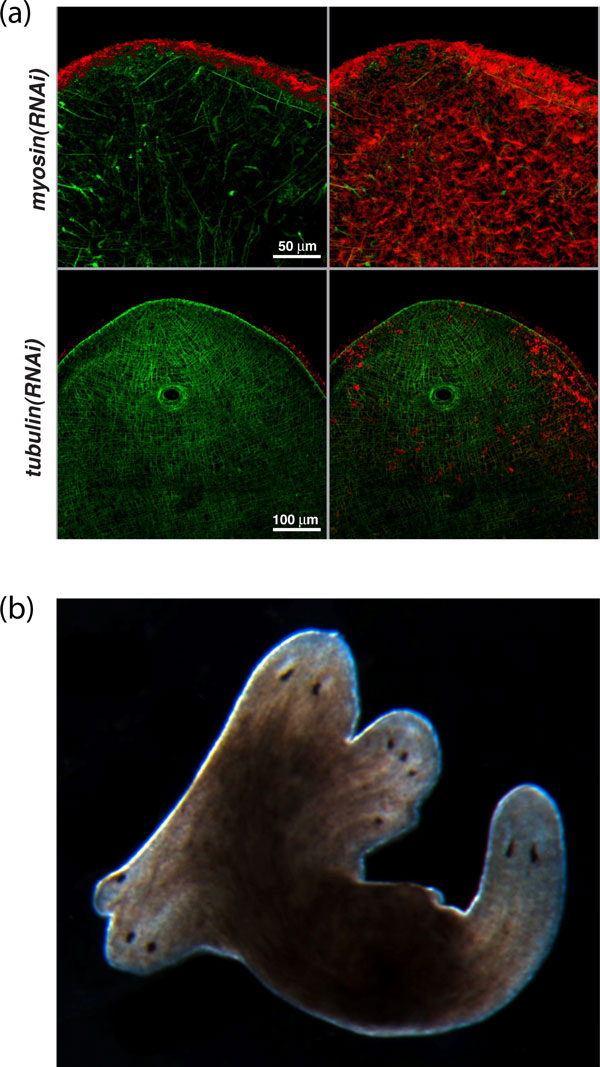
**Functional perturbation of gene function by RNA-mediated genetic interference (RNAi) in planarians**. **(a) **First RNAi effects reported in planarians show the specific loss of myosin (green) and tubulin (red) in regenerating tissues [23]. **(b) **Formation of multiple heads in an unamputated organism treated with β-*catenin(RNAi) *[25].

Yes. RNA interference (RNAi) can be used to robustly abrogate specific gene function [16], which became possible in 1998, when we extended to planarians Dr Andy Fire (our then downstairs neighbor at the Carnegie Institution for Science in Baltimore, MD) and Dr Craig Mello's discovery that double-stranded RNA could silence gene expression in *Caenorhabditis elegans*. We demonstrated the efficiency and specificity of this method in planarians by targeting and measuring the protein products of the myosin and tubulin genes (Figure 5a), which appeared in press a year later [23]. Presently, RNAi is the principal methodology being used by the planarian community to functionally interrogate genes and their functions in this organism. This method has allowed investigators to uncover remarkable phenotypes in RNAi-based screens [24] and signaling pathway perturbations (Figure 5b) [13,25].

## What lies ahead for planarians in particular and the field of regeneration in general?

Regeneration remains one of the last untamed frontiers of developmental biology. It is amongst the oldest biological problems known to humankind, dating back to antiquity in many cultures and, perplexingly, still awaiting a satisfactory mechanistic explanation. It is my firm belief that the time to plumb the molecular depths of regeneration is now. Tremendous strides have been made in the study of regeneration in Hydra, planarians, zebrafish, newts, and salamanders. Hence, a critical mass of knowledge is accruing that would permit a systematic interspecies comparison of regenerative capacities across very distant and diverse phyla. Equally important, a systematic and formal exploration of how the mechanisms of regeneration compare to embryogenesis can now begin in earnest. Such a comparison would help address the long-standing question of whether regeneration is simply a recapitulation of development or made possible by independent mechanistic innovations. In the case of planarians, are their embryonic stem cells functionally different from neoblasts? When during embryogenesis are neoblasts specified? To what extent are embryonic axes formation and organogenesis mechanisms similar or dissimilar between planarian embryogenesis and regeneration? Is the genetic toolkit required to organize body axes and facilitate organogenesis during embryogenesis the same as during regeneration? This is a particularly important question because many organisms such as the mouse, fruitfly, and frog can recover from ablation of numerous blastomeres or substantial injury to embryonic organs, yet display limited regenerative capacities as adults. Therefore, testing whether regulative development occurs in planarian embryos, for example, may help us identify key differences crucial to preserving regenerative abilities into adulthood. These and many more fascinating questions abound [8], so it is clear that when it comes to regeneration, we have but just begun to scratch the surface.
